# Immune reconstitution inflammatory syndrome associated with disseminated histoplasmosis and TNF-alpha inhibition

**DOI:** 10.1016/j.mmcr.2018.12.008

**Published:** 2018-12-22

**Authors:** Theodore Wright, Basak Coruh, David Fredricks, Nina Kim

**Affiliations:** a Division of Infectious Diseases, University of Washington, Harborview Medical Center, Seattle, WA 98104, United States; b Division of Pulmonary and Critical Care, University of Washington, University of Washington Medical Center, Seattle, WA 98104, United States; c Division of Infectious Diseases, University of Washington, Fred Hutchinson Cancer Research Center, Seattle, WA 98104, United States

**Keywords:** Immune reconstitution inflammatory syndrome, Histoplasmosis, TNF-alpha

## Abstract

Endemic fungal infections are a significant problem for patients on TNF-alpha inhibitors. Immune reconstitution inflammatory syndrome, IRIS, can present in patients with waning TNF-alpha inhibition and an endemic fungal infection. There may be a potential role that TNF-alpha inhibitors can play in mitigating IRIS related to disseminated endemic fungal infections.

## 1 Introduction

Modulation of inflammation through blockade of tumor necrosis factor (TNF)-alpha has been immensely successful in treating many autoimmune conditions. However, with this treatment comes an increasing incidence of opportunistic infections with endemic mycoses due to immunosuppression. In fact, histoplasmosis is estimated to be 3-fold more common than Mycobacterium tuberculosis in patients on TNF-alpha inhibitors in the United States [1]. There have also been emerging reports of immune reconstitution inflammatory syndrome (IRIS) in the context of these infections with use of TNF-alpha inhibitors. The rise in use of TNF-alpha inhibitors necessitates a better understanding of how best to address these adverse outcomes.

IRIS in patients with HIV and TB co-infection is the best described clinical variant although it remains unclear how best to manage this phenomenon and whether insights from these patients can be extrapolated to patients on TNF-alpha inhibition with invasive fungal infection. Treatment of IRIS can at times requires the administration of steroids. However, this approach is not always successful and there have been reports of patients who present with a severe inflammatory response during the treatment of TB or fungal infections that appear to be steroid-refractory and require other treatment options [2], [3], [4].

We describe here a case of a severe paradoxical inflammatory response during treatment of disseminated infection with *Histoplasma capsulatum* in a patient with Crohn's disease who was receiving TNF-alpha inhibition. This case highlights the potential role of reintroducing TNF-alpha inhibition in the treatment of steroid-refractory IRIS with disseminated histoplasmosis.

## 2 Case

This is a case of a 30 year-old gentleman with a history of Crohn's disease. He was on a regimen of infliximab, infused every 8 weeks and oral methotrexate daily. He had no other significant medical history. He was in his usual state of health until he developed a sore throat and fevers on day 0. His symptoms began while he was traveling in Europe. During his trip, he took a 10-day course of amoxicillin and felt some improvement.

After returning to the US he felt well but on day 14 he developed fevers and sore throat again. He was evaluated at an urgent care clinic and sent home with a diagnosis of a viral syndrome and instructed to treat this with non-steroidal anti-inflammatory agents. He felt some improvement initially but presented again on the day of admission with concern that he may not be well enough for his infusion of infliximab. He was found to have a temperature of 38.3 C and on day 30 was sent to the emergency department for further evaluation.

In the emergency department, he reported a non-productive cough, fevers, and sore throat. He had elevated liver enzymes: aspartate aminotransferase (AST) 340, alanine aminotransferase (ALT) 540, alkaline phosphatase 145. Additional testing was sent including a Monospot test, cytomegalovirus (CMV) and Epstein's Barr virus (EBV) serum viral levels, respiratory viral panel by PCR, adenovirus serum viral level, HIV antibody/antigen as well as HIV viral level, viral hepatitis serologic panel, human herpesvirus type 6 (HHV6) serum viral level, Varicella zoster virus (VZV) serum viral level, and syphilis IgG. He was admitted to the inpatient medicine service for fevers and hepatitis of unknown origin. His social history was remarkable for frequent trips to the Midwest for work. He did not pursue outdoors activity while traveling. He did not smoke, drink alcohol or use illicit drugs.

**Image 1:**
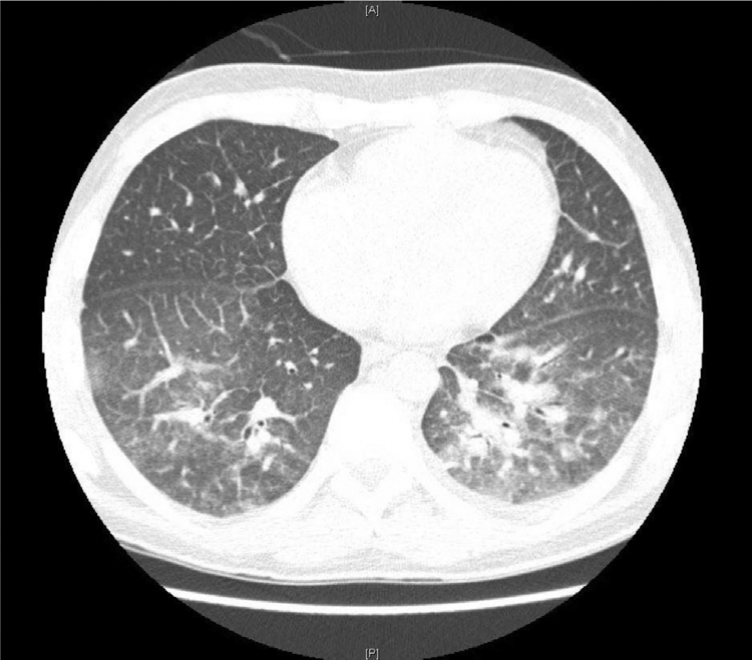
CT chest ordered for hypoxemia and showed ground-glass opacities in the lung bases.

**Image 2:**
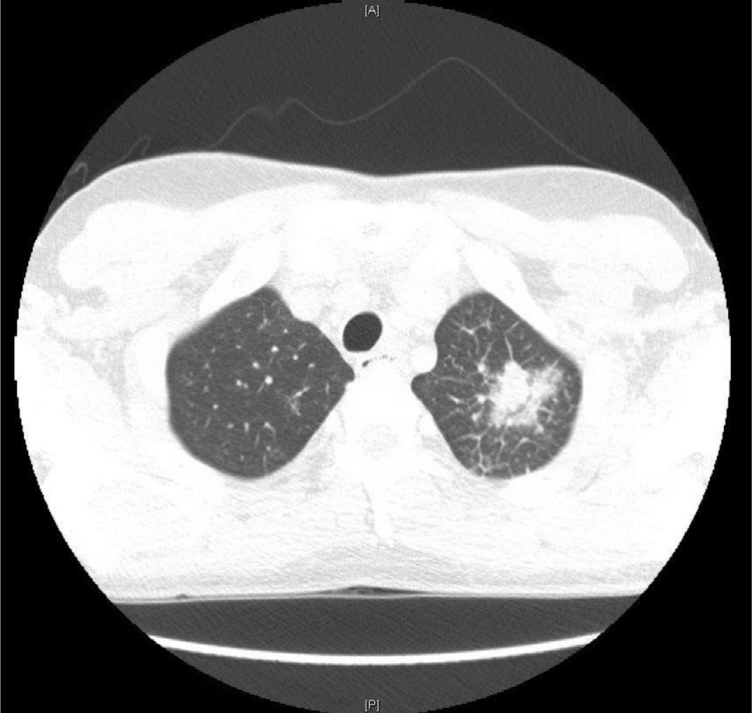
CT chest ordered for hypoxemia and showed a nodule in the left upper lobe.

On day 31 he developed daily fevers to 40 C and subsequently progressed to hypoxemic respiratory failure that required high-flow supplemental oxygen and transfer to the intensive care unit. He had a chest CT showing ground-glass opacities at the lung bases and a left upper lobe nodular opacity (Image 1, Image 2). His CT also demonstrated tonsillar enlargement and splenomegaly. He had a laryngoscopy performed that revealed an exudative pharyngitis. He was noted to have atypical lymphocytosis that peaked to 12,000 cells/uL on day 33. He was started on broad-spectrum antibiotics for presumed hospital-acquired pneumonia; including coverage of atypical organisms. He underwent a bronchoscopy on day 33. Bronchoalveolar lavage (BAL) fluid was negative for *Legionella* culture, AFB stain and culture, pneumocystis stain, and bacterial culture. Mycoplasma, zygomycetes and Mycobacterium tuberculosis were not detected by PCR. A respiratory viral panel by PCR from the lavage fluid was positive for Bocavirus and one of 2 samples for aspergillus galactomannan was moderately elevated but later determined to be negative on repeat testing.

On day 33 his AST peaked at 351 and his ALT at 662. His viral studies sent earlier returned negative. He had further serologic testing for atypical organisms including *Blastomyces*, *Coccidioides*, Q fever, *Bordetella pertussis*, as well as urine *Legionella* antigen that all returned negative. He was started on methylprednisolone and his fevers improved. His antibiotics were discontinued when his bacterial cultures were negative at 48 h. On day 37 the fungal PCR from BAL returned positive for *Histoplasma capsulatum* and cultures from that fluid later grew the organism. Urine *Histoplasma* antigen testing, sent earlier during his hospitalization, ultimately returned with a titer of 2.16 ng/mL (normal high 0.1 ng/mL). His *Coccidioides* antibody was also positive, but this was felt to be due to cross-reactivity from his Histoplasma infection.

On day 37 he was started on liposomal amphotericin B and 6 h later he had his highest fever to 42 C along with hypotension, tachycardia and worsening hypoxemia despite having received a dose of methylprednisolone earlier that day. He was felt to have a paradoxical worsening with treatment and exacerbation of immune reconstitution inflammatory state rather than an infusion reaction due to amphotericin given this time course [5]. His infliximab was restarted the following day with subsequent resolution of his fever, hemodynamic instability and improvement in his respiratory status within hours of this infusion. He continued amphotericin B for one week before transitioning to oral itraconazole. His ALT/AST improved significantly. He was discharged on oral itraconazole with continued clinical improvement and recovery as an outpatient.

## 3 Discussion

*Histoplasma capsulatum* is a dimorphic fungus that causes infection in both immunocompetent and immunocompromised individuals. It has worldwide endemicity but is most prevalent in the Americas, especially the midwestern states near the Ohio and Mississippi River valleys. The presentation of histoplasmosis can be very heterogeneous, making diagnosis challenging. Sites of involvement can include the pharynx, lung, liver, skin, bone marrow or brain. Although not considered typical sites of infection with Histoplasma, there are case reports of pharyngitis, epiglottitis, and necrotic tonsillitis due to histoplasmosis [6], [7].

Histoplasmosis begins with the inhalation of fungal spores that are then engulfed by pulmonary macrophages. This is followed by replication of the fungi within pulmonary macrophages; eventually stimulating a T-cell-mediated immune response through numerous cytokines [8]. In vitro data and clinical experience have shown that TNF-alpha is perhaps the most important of these regulatory cytokines. In vitro mouse data demonstrate that administration of a monoclonal antibody to TNF-alpha can increase the risk of disseminated infection with Histoplasma [9]. When TNF-alpha is inhibited in mice infected with M. tuberculosis, the accumulation of immune cells and the expression of vascular adhesion molecules are reduced, preventing the formation of granulomas [10]. Furthermore, blockade of TNF-alpha after granuloma formation contributed to the destruction of granulomas [10]. However, in addition to this, mouse models have also demonstrated improved clearance of tuberculosis when TNF-alpha is inhibited during treatment [11]. Mice treated for tuberculosis experienced a rise in TNF-alpha levels that potentially enabled slowly replicating mycobacteria to form granulomas and thereby evade the immune system. When these mice were also given etanercept, a soluble TNF-alpha receptor fusion protein, the rates of TB clearance were improved and rates of relapse were reduced [11].

Tumor Necrosis Factor (TNF)-alpha inhibitors bind to TNF, a key pro-inflammatory cytokine, and are known to inhibit T helper type 1 response, preventing the formation of granulomas and mitigating the inflammatory response in a variety of diseases [10]. A significant adverse effect of TNF antagonism is an increased risk for serious infection from endemic mycoses. Several reports have documented a paradoxical response to treatment of TB as well as histoplasmosis in patients who were on TNF-alpha inhibition and stop this therapy, and this is increasingly recognized as a form of immune reconstitution inflammatory syndrome (IRIS) [2], [3], [4], [12], [13], [14]. A surge of pathogen-specific interferon (IFN) gamma and non-specific TNF alpha production can occur when TNF alpha inhibitor levels wane in the setting of a concomitant infection with histoplasmosis, and can present as both an unmasking phenomenon or IRIS in the setting of known infection [12].

Immune Reconstitution Inflammatory Syndrome, or IRIS, was first described as a consequence of initiating antiretroviral therapy in patients with HIV and concurrent opportunistic infection but has been increasingly recognized as a clinical syndrome that can occur when weaning or discontinuing immunosuppression in patients without HIV who are on TNF-alpha inhibitors or corticosteroids. When this immunosuppression is reduced in the setting of a concomitant infection, an exuberant response in the form of pathogen-specific inflammation can occur [15].

There is some clinical evidence for the role of TNF alpha blockers in the treatment of IRIS in HIV-infected patients, including a series of three HIV-infected patients with tuberculosis who presented with steroid-refractory IRIS and improved with initiation of TNF-alpha inhibitors [2] but our report is the first to demonstrate a possible role in the setting of disseminated histoplasmosis. IRIS has been treated with steroids with some success but this would not be expected to treat the root cause, rebound of Th1 activity, as precisely as a TNF-alpha inhibitor. Restarting TNF alpha inhibitors would, in contrast, address the underlying cause of this surge of inflammation with more precision. Another report noted clinical success with TNF-alpha inhibition in the treatment of steroid-dependent IRIS in patients with HIV infection and pulmonary tuberculosis [3]. It is common to withhold TNF-alpha blockers in patients presenting with an opportunistic infection [12], such as histoplasmosis, but growing evidence suggests it can be reinitiated early in the course of infection without significant sequelae, and perhaps serve as a useful adjunctive therapy for severe IRIS. This is demonstrated in a case series of 98 patients with histoplasmosis who were on TNF-alpha inhibitors [16]. In this case series, 3 patients were continued on their TNF-alpha inhibitor and were successfully treated for histoplasmosis.

Our patient presented with hypoxemic respiratory failure, fevers, and transaminitis 8 weeks after his last infliximab treatment; all consistent with IRIS. He had a paradoxical reaction to treatment of his histoplasmosis with severe fevers and worsening hypotension several hours after his amphotericin infusion. He improved rapidly with re-introduction of his TNF-alpha inhibitor and continued amphotericin. The single case nature of our report with lack of controls as well as our inability to establish a causal association with TNF-alpha reintroduction and clinical improvement are notable limitations but the prompt change in his fever curve and clinical status shortly after infliximab was remarkable and difficult to ignore. Our case illustrates the risk of severe IRIS in patients with concomitant Histoplasma infection, the heterogeneity of their presentation, and the potential role of TNF inhibitors in the treatment of IRIS in this setting.
